# A case report of primary prostate intravascular large B-cell lymphoma

**DOI:** 10.1097/MD.0000000000049834

**Published:** 2026-07-17

**Authors:** Fengju Yuan, Qian Liu, Wuwu Ding, Jia Nie, Xianwei Wang

**Affiliations:** a Department of Pathology, Deyang People’s Hospital, Deyang, Sichuan, China.

**Keywords:** diagnosis, immunohistochemistry, intravascular large B-cell lymphoma, prostate

## Abstract

**Rationale:**

Primary intravascular large B-cell lymphoma (IVLBCL) is a rare and aggressive extranodal lymphoma that rarely affects the prostate. Its nonspecific clinical and laboratory features often lead to misdiagnosis as benign prostatic hyperplasia (BPH) or prostatitis, delaying appropriate treatment. We report a case of primary prostatic IVLBCL initially misdiagnosed as BPH, highlighting the diagnostic challenges and the importance of comprehensive pathological evaluation.

**Patient concerns:**

A 75-year-old male presented with a 1-year history of a weakened urinary stream, dribbling, increased nocturia, and urinary urgency. Symptoms transiently improved with self-medication of the α1-blocker tamsulosin but later recurred.

**Diagnoses:**

Histopathological examination revealed clusters of atypical tumor cells within the prostatic vasculature. Immunohistochemistry showed positivity for leukocyte common antigen, Vimentin, CD20, and CD79a, with a Ki-67 index > 90%. A final diagnosis of primary intravascular large B-cell lymphoma of the prostate was established.

**Interventions:**

Following diagnosis, the patient and his family declined any antitumor therapy (including chemotherapy) and opted for best supportive care and were discharged against medical advice.

**Outcomes:**

The patient died 5 months after diagnosis without receiving any subsequent antitumor therapy.

**Lessons:**

Prostatic IVLBCL is a diagnostic mimic of BPH and requires a high index of suspicion. Immunohistochemistry and molecular studies are essential for accurate diagnosis. Early recognition and appropriate chemotherapy can improve outcomes in this rare malignancy.

## 1. Introduction

Intravascular large B-cell lymphoma (IVLBCL) is a rare form of non-Hodgkin lymphoma with an incidence rate of only 0.095 per 100,000 individuals.^[1]^ According to the 2022 fifth edition of the World Health Organization classification of lymphoid neoplasms, IVLBCL is classified as a large B-cell lymphoma. It can be categorized into several subtypes: the classical subtype, the cutaneous variant subtype, and the hemophagocytic lymphohistiocytosis-related subtype, each characterized by distinct clinical presentations, pathological features, and prognoses.^[2]^ Typically, IVLBCL manifests in the skin and central nervous system (CNS), although it may also affect the lungs, kidneys, and other organs.^[3]^ Primary IVLBCL of the prostate is particularly uncommon and presents with nonspecific symptoms that complicate accurate clinical and pathological diagnosis. This paper reports a case of prostatic IVLBCL that initially presented as prostatic hyperplasia. It examines the clinical and pathological characteristics of prostatic IVLBCL and outlines the corresponding treatment approaches. The discussion includes a review of pertinent literature, aiming to enhance the understanding of this condition among clinicians and pathologists to facilitate timely and effective diagnosis and treatment, thereby improving patient outcomes.

## 2. Case

**Figure 1.**
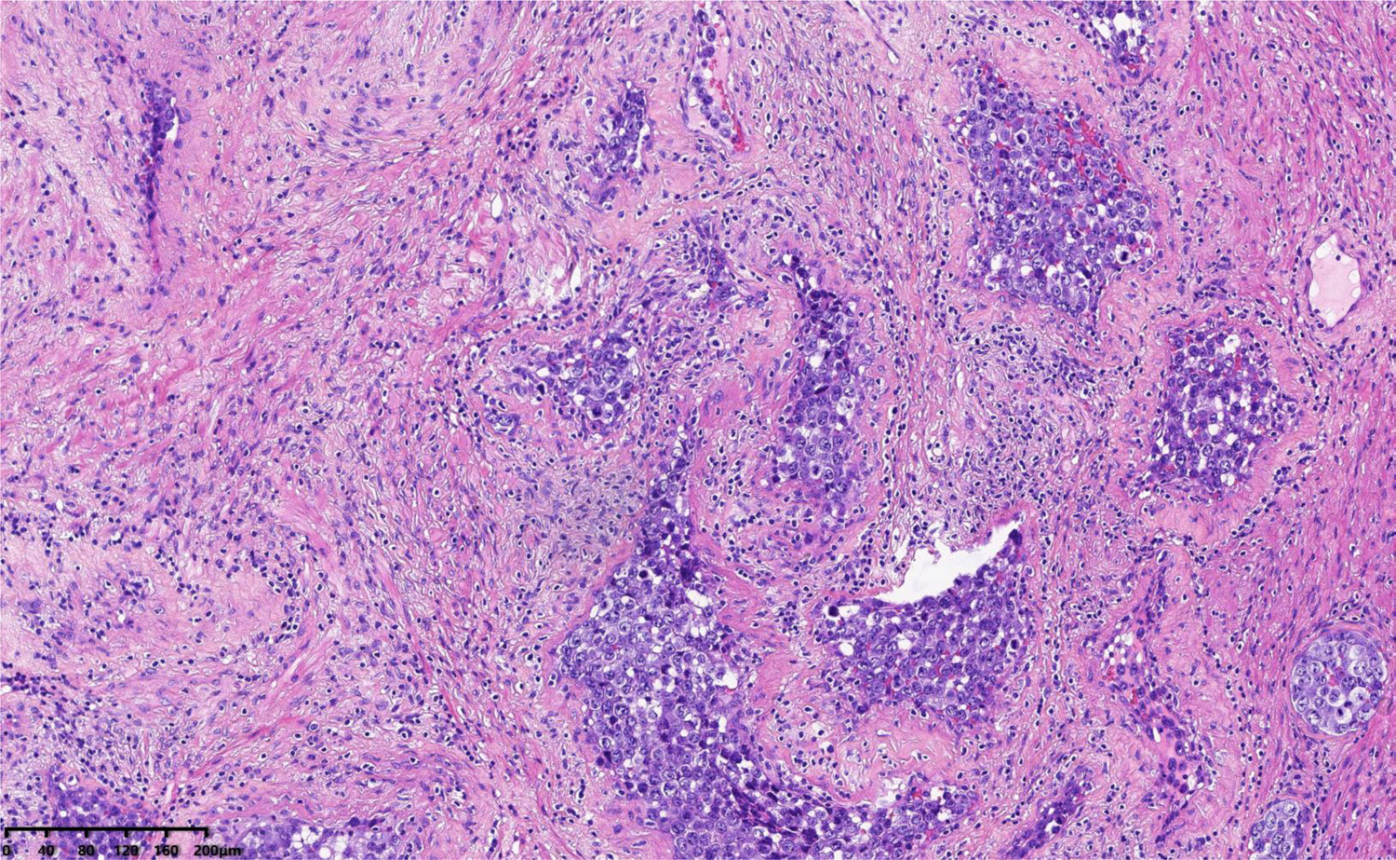
At 100x magnification with hematoxylin and eosin staining, tumor cells are seen clustered within blood vessels. These cells range from medium to large, with an increased nuclear-to-cytoplasmic ratio. The nuclei are large, round, and contain clearly visible nucleoli.

**Figure 2.**
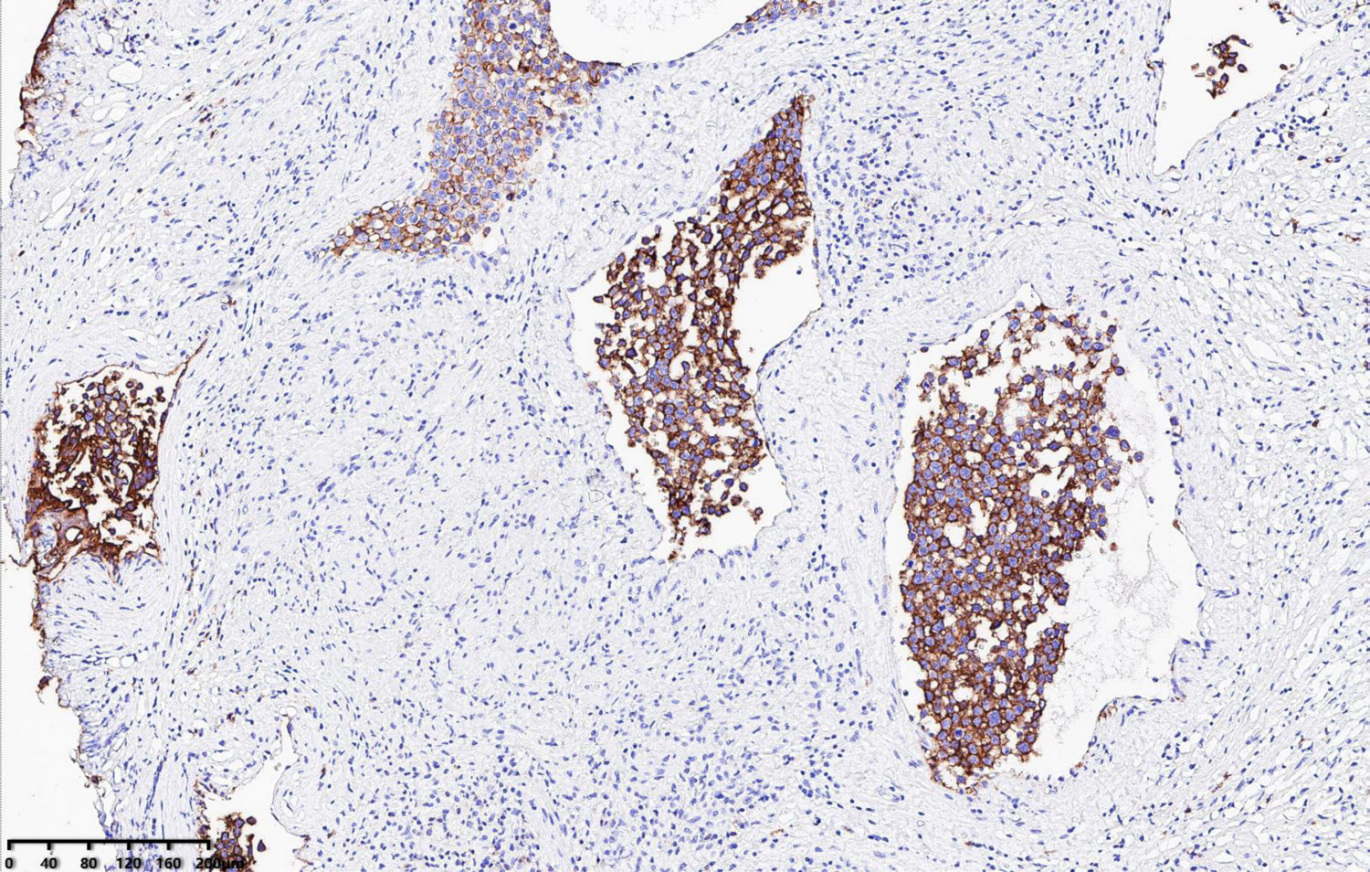
Tumor cells were positive for CD20, EnVision method.

**Figure 3.**
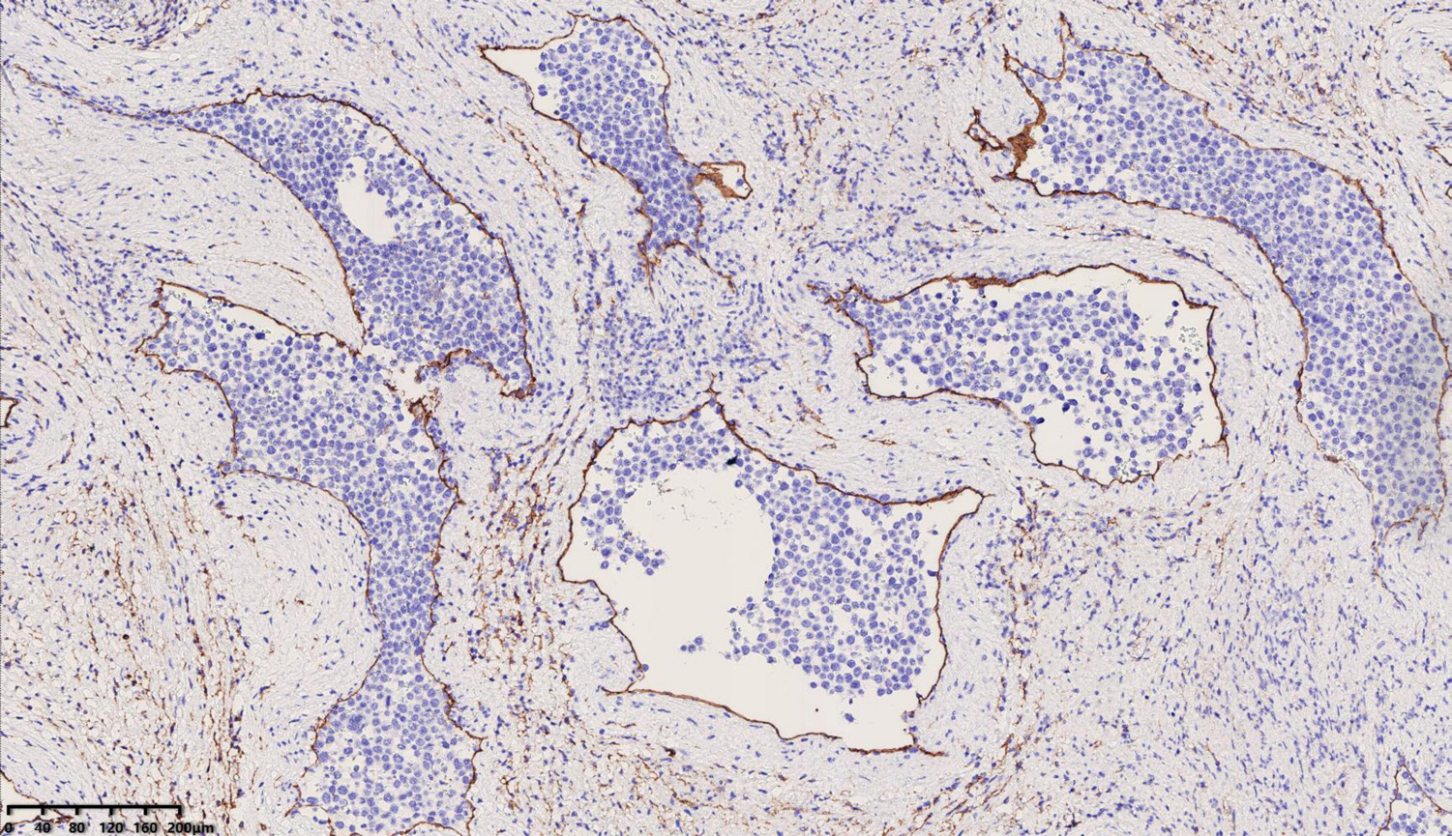
CD34 staining was positive for blood vessels, confirming that tumor cells were located within these structures.

A 75-year-old male presented with symptoms including a weakened urinary stream, dribbling, increased frequency of nocturia, and urgent urination that had persisted for over a year without a clear cause. The symptoms initially improved after self-medicationwith the α1-blocker tamsulosin but later returned. Upon seeking further medical help, he was diagnosed with “prostatic hyperplasia” in an outpatient setting. Physical examination revealed no palpable abnormalities in the liver, spleen, or lymph nodes. Digital rectal examination showed an enlarged, firm prostate with a shallow central sulcus and no palpable nodules. A urinary color Doppler ultrasound revealed prostatic enlargement to 5.7 cm by 5.1 cm with an irregular shape and a 2.1 cm protrusion into the bladder, along with a consistent echo texture throughout the parenchyma. Laboratory tests indicated the following: red blood cells at 3.6 × 10^12^/L (decreased), hemoglobin at 108 g/L (decreased), hematocrit at 31.8% (decreased), platelets at 115 × 10^9^/L (decreased), elevated total prostate-specific antigen (PSA) at 6.07 ng/ml, decreased albumin (at 38.4 g/L, increased globulin at 41.4 g/L, and an albumin/globulin ratio of 0.93 (decreased). Lactate dehydrogenase (LDH) at 603 (units per liter; increased), β_2_-microglobulin at 4.34 mg/L (increased); elevated levels of C-reactive protein at 10.90 mg/L, interleukin (IL)-6 at 21.06 pg/ml, IL-10 at 169.60 pg/ml, and IL-12 at 4.45 pg/ml were also found. Histopathological examination showed normal prostate gland morphology, but there were clusters of tumor cells within the stroma, predominantly located within blood vessels. These cells demonstrated poor cohesion, significant cellular atypia, medium to large size, an elevated nuclear-to-cytoplasmic ratio, scant cytoplasm, large and round nuclei, prominent nucleoli, and high mitotic activity (Fig. 1). Immunohistochemical tests revealed positive staining for leukocyte common antigen, Vimentin, CD20 (Fig. 2), and CD79a, with a Ki-67 proliferation index exceeding 90%. CD34 staining verified the intravascular location of all tumor cells (Fig. 3). The cells were negative for CD3, CD5, CD10, Bcl-6, MUM1, CD30, Bcl-2, C-MYC, CD117, SALL-4, PLAP, OCT3/4, GATA-3, CK7, CK20, P63, P504S, PSA, AR, CK, and CK5/6. EBER 1/2-ISH also showed no evidence of Epstein–Barr virus. Based on the combined histological and immunohistochemical findings, the diagnosis was confirmed to be primary IVLBCL of the prostate. After the diagnosis was established, the patient opted against all forms of antitumor therapy, receiving only best supportive care before leaving the hospital against medical advice. He subsequently died of the disease 5 months later.

## 3. Discussion

The incidence of IVLBCL is extremely rare, accounting for < 1% of all non-Hodgkin lymphomas. Cases of IVLBCL occurring in the prostate are even rarer. Our literature review identified only 11 reported cases of IVLBCL in the prostate, all of which were documented as case reports.^[4–14]^ The scarcity of this disease and its nonspecific symptoms often lead to misdiagnosis or delayed diagnosis, hindering timely and optimal treatment. Therefore, a thorough understanding of the clinicopathological features of prostate IVLBCL is essential for ensuring an early and accurate diagnosis, providing a basis for developing more effective treatments, and ultimately improving patient outcomes in terms of survival and quality of life.

Research indicates^[3,15–18]^ that IVLBCL primarily affects middle-aged and elderly individuals, with typical onset between 60 and 70 years. Clinically, it often presents with unexplained fevers, various nonspecific neurological symptoms, skin rashes, and a lack of significant lymph node enlargement. Common laboratory abnormalities include anemia, thrombocytopenia, leukopenia, a low erythrocyte sedimentation rate, and an elevated monocyte count. Additionally, increased levels of LDH, C-reactive protein, β_2_-microglobulin, and albumin are frequently noted. The clinicopathological characteristics of prostate IVLBCL align with those of other manifestations of IVLBCL. In our review of 12 cases of prostate IVLBCL, patients ranged in age from 60 to 79 years old, with a median age of 71 years. Clinically, none of the patients with prostate IVLBCL showed significant lymphadenopathy. Only a few patients reported fever of unknown origin (3 out of 12) or neurological symptoms (2 out of 12), and no cases of skin rashes were documented. Notably, 91.67% of the patients (11 out of 12) initially sought medical help for symptoms of urinary tract obstruction, which were initially misdiagnosed as benign prostatic hyperplasia (BPH). Laboratory tests for those diagnosed with IVLBCL commonly show traditional hematological abnormalities and elevated levels of LDH, among other markers. In our review, an increase in β_2_-microglobulin was also noted. Uniquely, a small group of patients (4 of 14) with primary prostate IVLBCL displayed a slight increase in PSA levels. Additionally, our case identified elevated levels of IL-6, IL-10, and IL-12. Of particular interest, our patient’s serum IL-10 level was 169.60 pg/mL. This exceeds the previously proposed diagnostic threshold of 65 ng/L (equivalent to 65 pg/mL) for IVLBCL screening, which has been associated with sensitivity and specificity rates of up to 80% and 100%, respectively.^[19]^ The markedly elevated IL-10 in this case further supports its potential utility as a valuable biomarker for IVLBCL, even in this unusual prostatic presentation. Existing literature indicates^[20]^ that serum cytokines are integral to the pathogenesis of hematologic malignancies. Specifically, IL-10 can promote tumor cell growth and immune evasion by altering the immunosuppressive environment, suggesting its potential as a valuable biomarker for IVLBCL screening.

Although the aforementioned clinical and laboratory features have certain suggestive value, the definitive diagnosis of primary prostatic IVLBCL remains a major challenge. Unusual localization and atypical presentations frequently delay diagnosis, necessitating a low threshold for biopsy. In the present case, lower urinary tract symptoms closely mimicked BPH, with no discrete nodule or mass. Reliance on conventional biopsy indications (elevated PSA or suspicious nodules) may therefore delay the diagnosis of rare prostatic lymphomas. Evolving prostate biopsy strategies are relevant to this diagnostic gap. Current screening protocols often omit systematic biopsies in favor of magnetic resonance imaging‑targeted cores to reduce overdiagnosis of indolent prostate cancer.^[21,22]^ However, such streamlined approaches may miss diffuse or intravascular malignancies (e.g., IVLBCL) that lack a radiologically visible lesion. Therefore, in patients with persistent unexplained urinary symptoms, elevated LDH, cytopenias, or inflammatory markers (even without a visible lesion) a lower threshold for systematic prostate biopsy or deeper sectioning is warranted. Once a biopsy specimen has been obtained, the key to accurately identifying IVLBCL lies in familiarity with its distinctive histopathological and immunohistochemical characteristics. The histopathological characteristics of prostatic IVLBCL align with those observed in IVLBCL affecting other parts of the body. Tumor cells are primarily found within blood vessels, usually within small to medium-sized vessels with thin walls, although they can sometimes appear in larger vessels as well. These cells tend to proliferate inside the vessel lumen and remain close to the vessel wall, rarely invading surrounding tissues. Minimal extravascular invasion was observed in some cases. The tumor cells vary in appearance, including centroblastic, immunoblastic, or plasmablastic types, all showing significant atypia. They are typically medium to large, with a high nuclear-to-cytoplasmic ratio, large round nuclei, prominent nucleoli, and frequent mitotic activity. IVLBCL is classified as a subtype of large B-cell lymphoma, known to predominantly express B-cell markers such as CD19, CD20, CD79α, and PAX5, while generally lacking T-cell markers like CD3 and CD5.^[3,15]^ In our study, CD20 was present in all examined cases of prostate IVLBCL, and CD3 tests were negative in all 12 cases. Although the tumor in our patient was CD5-negative, CD5 expression has occasionally been reported in IVLBCL.^[3,23]^ Additionally, the diagnostic process for IVLBCL includes evaluating markers such as CD31, CD34, CD10, bcl-6, and MUM1. CD31 and CD34 are especially crucial for identifying blood vessel walls, which helps confirm the intravascular nature of the tumor. Based on the expression of CD10, bcl-6, and MUM1, IVLBCL can be classified into germinal center and non-germinal center origins, with most cases being of non-germinal center origin. For pathologists examining routine transurethral resection of the prostate or needle biopsy specimens initially obtained with a benign clinical impression, certain histological clues should raise suspicion for IVLBCL: the presence of atypical lymphoid cells exclusively or predominantly within vascular lumina, even when the overall architecture appears preserved; an apparent “increase” in intravascular cellularity that contrasts with relatively normal intervening parenchyma; and the finding of large, dyshesive cells with high nuclear-to-cytoplasmic ratios and prominent nucleoli confined to vessel spaces. When these features are observed, immunohistochemical staining for CD20, CD79a or PAX5, CD3, CD34, and CD31 is essential to confirm the intravascular B-cell nature of the proliferation and to avoid misinterpretation as reactive endothelial change, carcinoma emboli, or other intravascular processes. More broadly, this case reinforces a central principle in prostate pathology: the diagnostic value of biopsy material depends on careful morphological assessment, adequate sampling, clinicopathological correlation, and the judicious use of ancillary tests.^[24]^IVLBCL is a highly aggressive cancer with a generally poor prognosis. The key factors that contribute to worse outcomes in IVLBCL patients include high LDH levels (≥ 700 units per liter), involvement of the CNS, and hemophagocytic syndrome.^[25]^ The scarcity of IVLBCL cases has precluded large-scale clinical trials, leading to the absence of a standardized treatment approach. Generally, treatment follows the R-CHOP protocol (rituximab, cyclophosphamide, doxorubicin, vincristine, and prednisone), which is the standard regimen for diffuse large B-cell lymphoma. Prior studies have indicated^,[23,25,26]^ that combining rituximab with CHOP can significantly improve the complete response rate, event-free survival, and overall survival for patients with IVLBCL, leading to a significantly higher 5-year survival rate compared to other treatments. A phase 2, multicenter, single-arm trial in Japan found that R-CHOP combined with high-dose methotrexate is an effective initial treatment for IVLBCL patients without CNS involvement, achieving a 2-year progression-free survival rate of 76% (95% confidence interval 58–87).^[27]^ Additionally, the high occurrence of MYD88 and CD79B mutations in IVLBCL patients suggests that adding Bruton tyrosine kinase inhibitors to the R-CHOP regimen could be a promising treatment strategy.^[28]^ A retrospective study by Kato K found that high-dose chemotherapy followed by autologous hematopoietic stem cell transplantation significantly improved the prognosis of patients.^[29]^ However, more clinical evidence is needed to confirm the effectiveness of these treatments. Currently, the treatment strategy for primary prostate IVLBCL follows the same protocol as that for IVLBCL affecting other body sites, primarily using the R-CHOP regimen. In our case series, all 5 patients treated with an R-CHOP-based regimen achieved complete remission. Notably, 1 patient who received both R-CHOP and radiotherapy maintained complete remission for 49 months and continues to be in good health without any signs of disease recurrence.

In summary, the diagnostic journey of this case underscores a critical lesson: for elderly males presenting with lower urinary tract symptoms that are atypical of BPH or who respond unexpectedly to conventional medical therapy, a high index of suspicion for rare neoplastic conditions, including IVLBCL, should be maintained. This is particularly true when laboratory findings also provide important clues, such as markedly elevated LDH, β_2_‑microglobulin, and IL‑10, along with anemia, thrombocytopenia, and mildly elevated PSA. Although these abnormalities are nonspecific, they carry important diagnostic value when combined with an atypical clinical presentation. Early recognition, coupled with timely biopsy and comprehensive immunohistochemical characterization, is essential to avoid diagnostic delay and to enable prompt initiation of appropriate therapy. The poor outcome of our patient, who declined chemotherapy and died 5 months after diagnosis, further highlights the aggressive nature of this disease and the importance of early and accurate diagnosis.

## Author contributions

**Conceptualization:** Fengju Yuan.

**Data curation:** Wuwu Ding.

**Funding acquisition:** Xianwei Wang.

**Investigation:** Fengju Yuan, Qian Liu.

**Methodology:** Fengju Yuan.

**Resources:** Wuwu Ding.

**Supervision:** Jia Nie.

**Validation:** Jia Nie.

**Writing – original draft:** Fengju Yuan, Qian Liu.

**Writing – review & editing:** Xianwei Wang.
